# Compressive Response and Damage Distribution of Fiber-Reinforced Concrete with Various Saturation Degrees

**DOI:** 10.3390/ma18071555

**Published:** 2025-03-29

**Authors:** Lu Feng, Xudong Chen

**Affiliations:** 1 College of Civil and Transportation Engineering, Hohai University, Nanjing 210024, China; lufeng980509@hotmail.com; 2 Department of Civil and Environmental Engineering, Politecnico di Milano, 20133 Milan, Italy

**Keywords:** fiber-reinforced concrete, cyclic loading, water-weakening effect, acoustic emission, damage constitutive model

## Abstract

Tunnels frequently experience issues such as lining spalling and water leakage, making the stability of tunnel support critical for engineering safety. Given that tunnels are subjected to various ground stress disturbances and groundwater influences, it is essential to investigate the mechanical properties and damage mechanisms of tunnel support materials under different loading paths and saturation levels. Fiber-reinforced concrete (FRC) is widely used for tunnel support; in this study, uniaxial compression tests were conducted on FRC with different fiber contents (0%, 0.5%, 1.0%) under varying loading paths (monotonic, pre-peak cyclic loading, full cyclic loading). The stress–strain behavior, volumetric strain, and elastic modulus were analyzed. The results indicate that increasing fiber content enhances strength and stiffness, while higher water content leads to a significant water-weakening effect, reducing both parameters. To classify crack types, the logistic regression (LR) algorithm is employed based on the AF-RA features, identifying tensile damage (which accounts for 60–80%) as more dominant than shear damage. Using this classification, AE event distributions reveal the spatial characteristics of internal damage in FRC. Gaussian process regression (GPR) is further applied to predict the AE parameters, enabling the assessment of the tensile and shear damage responses in FRC. The location and magnitude of the predicted wave crest indicate extreme damage levels, which become more pronounced under a higher saturation condition. A damage constitutive model is proposed to characterize the post-peak softening behavior of FRC. The numerical verification demonstrates good agreement with the experimental results, confirming the model’s capability to describe the softening behavior of FRC under various fiber and water contents.

## 1. Introduction

The spatial distribution of water resources often does not align with regional economic development and population distribution, posing challenges for the sustainable growth of water-scarce regions. Water transfer tunnels play a crucial role in transporting water over long distances. However, these tunnels are often deeply buried underground, making them susceptible to ground stress disturbances and groundwater infiltration. These factors can lead to tunnel lining damage and water leakage, ultimately compromising the structural integrity and overall performance of the tunnel system. Fiber-reinforced concrete (FRC) is widely used for tunnel support, necessitating a comprehensive analysis of its mechanical properties and water-weakening effects.

In practical engineering applications, concrete structures are subjected to various loading conditions, including compression, tension, bending, shear, and torsion. The cracking and damage mechanisms of concrete under different loading types have been extensively studied [1,2,3,4]. Golewski [2] investigated the fracture properties of different concrete composites by incorporating varying amounts of fly ash and nano-silica as additives to replace cementitious binders, and found that an optimal combination of mineral admixtures can effectively enhance concrete strength while reducing carbon emissions. Ribeiro et al. [3] examined the shear strength of precast concrete under different influencing factors, concluding that increasing the section height significantly enhances the shear resistance. Among these, the existing research on FRC covers several key areas: (1) Preparation techniques involve designing concrete mixtures with varying raw materials, mix ratios, and mineral admixtures to optimize the workability, mechanical properties, microstructure, and cost efficiency for different applications [5,6,7]. (2) Mechanical behavior and durability are analyzed through experimental studies, including uniaxial compression, direct tension, splitting, fracture, high-temperature exposure, and freeze–thaw tests, to understand the failure process and degradation mechanisms [8,9,10,11,12]. (3) Structural performance is investigated via axial compression, fatigue, shrinkage, creep, and bond-slip tests on FRC columns, leading to the establishment of constitutive models for FRC under fatigue loading and insights into the effects of fibers on the strain inhibition, shrinkage reduction, and bonding behavior between FRC and ordinary concrete [13,14,15]. (4) Fiber–cement matrix bonding characteristics are examined through direct fiber pull-out tests, revealing how the fiber type, end shape, surface roughness, and other factors influence the interfacial bond strength and the bridging role of fibers in concrete [16,17,18]. Additionally, some studies have explored the fatigue behavior of FRC under compressive loading. Medeiros et al. [19] analyzed the effects of loading frequency on the fatigue performance of both plain concrete and FRC. Poveda et al. [20] investigated the influence of the fiber content on fatigue life and proposed a fatigue damage criterion. Li et al. [21] examined the mechanical response and AE activity of ultra-high-performance concrete (UHPC) with varying steel fiber contents under uniaxial monotonic and cyclic loading, leading to a damage evolution law and a semi-empirical elastoplastic damage constitutive model. These studies highlight that fibers significantly improve the compressive performance of concrete, reducing the plastic strain accumulation and delaying elastic stiffness degradation. Despite these advancements, limited research deals with the water-weakening effect on FRC. Some studies have investigated the capillary water absorption and permeability of FRC [22]. Wang et al. [23] found that the water content and strain rate significantly influence the dynamic compressive strength and failure modes.

Non-destructive monitoring techniques enable the assessment of internal characteristic changes in specimens without causing damage, and have been widely applied to monitor the crack propagation and damage evolution in concrete materials. Various methods have been employed by researchers for such evaluations, including CT scanning, three-dimensional laser scanning, electro-mechanical impedance, nuclear magnetic resonance (NMR), acoustic emission (AE), and scanning electron microscopy (SEM). For example, Stamati et al. [24] utilized CT scanning to capture the heterogeneous mesostructure of concrete, and subsequently established a three-dimensional finite element mesoscopic model with enhanced discontinuity features to conduct uniaxial tensile numerical simulations. Sadowski et al. [25] employed three-dimensional laser scanning to analyze the surface morphology parameters of concrete. Naoum et al. [26] adopted a novel electro-mechanical impedance (EMI) technique to monitor concrete columns subjected to flexural loading, demonstrating that this method enables the quantitative and reliable observation of damage and cracking. Meanwhile, van der Heijden et al. [27] utilized NMR to investigate water migration in heated concrete samples. In this study, acoustic emission (AE) is selected as the monitoring method due to its operational convenience and relatively low cost, which can dynamically track the crack propagation and damage evolution in concrete. Tayfur et al. [28] used AE analysis, principal component analysis, and k-means clustering to characterize the three-point bending fracture process of steel FRC and the debonding behavior between the fibers and the cement matrix. Similarly, Chen et al. [29] proposed a supervised k-nearest neighbor (KNN) algorithm combined with AE parameters to effectively identify damage in the FRC matrix and fiber–matrix debonding. Guo et al. [30] introduced the spatial *b*-value to visualize the internal damage in fiber-reinforced polymer (FRP)-reinforced concrete cylinders using AE parameters. Li et al. [31] characterized the FRP reinforcement debonding in pull-out tests, analyzing the AE impact number, amplitude, and peak frequency while incorporating finite element stress field simulations.

From this literature review, it is evident that while extensive research has been conducted on the preparation, mechanical properties, structural performance, and fiber-bonding characteristics of FRC, as well as its engineering applications and economic feasibility, most studies focus on static loading conditions, particularly in shotcrete applications. However, as a key component of tunnel linings, the cyclic compression behavior of shotcrete under complex environmental disturbances, such as fluctuating ground stress, remains underexplored. Additionally, the damage evolution mechanism and post-peak constitutive model for polypropylene fiber-reinforced shotcrete under different cyclic loading paths have yet to be established. The existing AE research has rarely addressed the identification of cyclic compression damage in shotcrete.

In summary, given that the mechanical properties and damage mechanism of FRC under different types of ground stress disturbance and groundwater are still unclear, it is necessary to investigate the mechanical properties of FRC under various loading paths and the influence of water content on these properties. By integrating an AE analysis, the study provides a deeper understanding of the effects of the fiber and saturation level on the damage processes. The damage distribution mechanism is evaluated based on the AE parameters, with the logistic regression algorithm applied to differentiate the AF-RA distributions for the damage classification of AE events. Additionally, Gaussian process regression (GPR) is employed to predict the AE parameters. Finally, a damage constitutive model for FRC is established and validated.

## 2. Raw Materials and Test Scheme

### 2.1. Raw Materials

**Figure 1:**
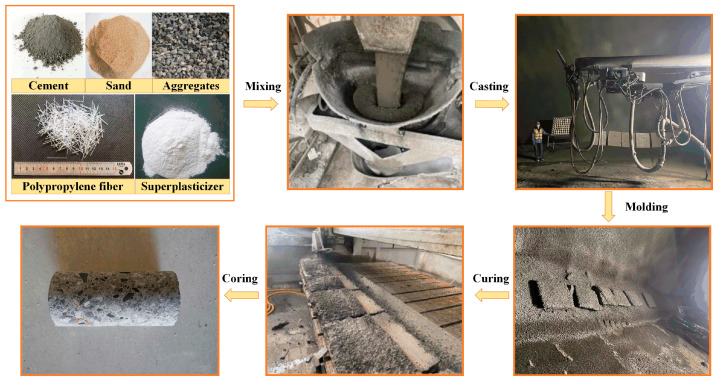
Flowchart for preparation of FRC.

Compared to ordinary concrete, FRC features smaller aggregate sizes and a higher sand content. The raw materials included P·O 42.5 Portland cement, river sand with a fineness modulus of 2.3, 5–10 mm limestone aggregates, water, a superplasticizer, and polypropylene fiber. The FRC specimens were prepared with fiber volume fractions of 0%, 0.5%, and 1.0%, and the mix proportions are listed in Table 1. The slump values for FRC-0, FRC-0.5%, and FRC-1.0% are 205 mm, 182 mm, and 170 mm, respectively. After 28 days of standard curing, the FRC slabs were cored, and the uniaxial compression specimens were fabricated into cylinders with a diameter of 50 mm and a height of 100 mm. A flowchart for the preparation of the specimens is shown in Figure 1.

The properties of the polypropylene fiber are listed in Table 2.

The properties of the superplasticizer are listed in Table 3.

The properties of the cement are listed in Table 4.

The coarse aggregate used was crushed limestone, with its physical properties summarized in Table 5. The particle size ranged from 5 to 10 mm, and the saturated compressive strength of the limestone was 97.8 MPa.

### 2.2. Experimental Program

**Figure 2:**
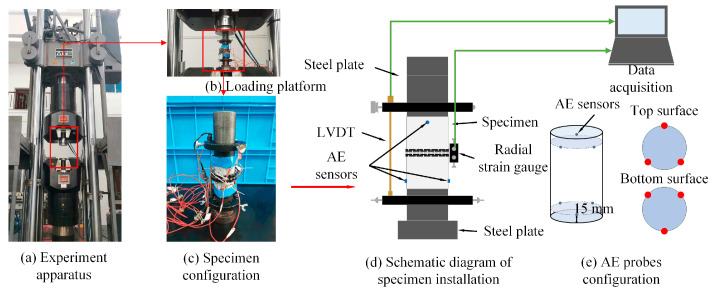
Experimental apparatus.

Monotonic and cyclic uniaxial compression experiments under different loading paths were conducted using an MTS Hydraulic Grip testing system (Figure 2). This apparatus has upper loading limits of 2500 kN and 2700 kN for dynamic and static tests, respectively, and a maximum confining pressure of 100 MPa. It maintains a stable confining pressure during loading through self-compensation technology. Axial strain was measured using a linear variable displacement transducer (LVDT), while radial strain was recorded using a strain gauge. In the uniaxial compression test, the LVDT directly measures the deformation from the top to the bottom surface of the specimen, representing the total axial displacement (mm). The axial strain is determined through the ratio of the axial displacement to the specimen length. As the elastic modulus of steel is significantly higher than that of concrete, the axial strain in the steel plates is negligible during data processing.

Real-time AE monitoring was performed using the SAMOS™ detection system, developed by PAC corporation in State of New Jersey of the United States. The monitoring system was configured with a threshold of 35 dB and a filtering frequency range of 1–60 kHz. Six AE probes were positioned at 120° intervals around the specimen, 15 mm from its ends (Figure 2d). To minimize the noise interference, a high-vacuum grease was applied between the AE probes and the specimen surface.

**Figure 3:**
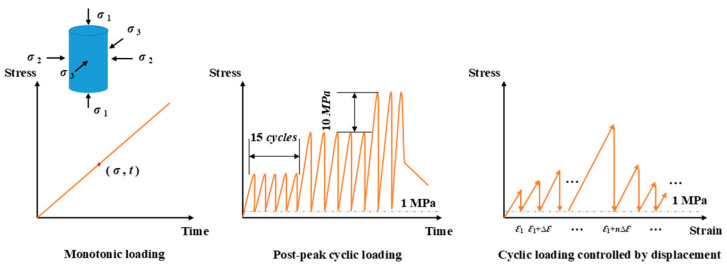
The diagram of the three loading paths.

The experimental program consisted of three loading paths: (1) Monotonic loading: Controlled by axial displacement at a rate of 0.1 mm/min, with loading continued until the post-peak residual stage. (2) Pre-peak cyclic loading: Controlled by axial stress in a sinusoidal waveform, with an incremental stepwise cyclic loading rate of 0.05 MPa/s and a frequency of 0.2 Hz. The upper stress limit increased by 10 MPa every 15 cycles, while the unloading lower limit remained at 1 MPa. (3) Full cyclic loading: Controlled by axial displacement, with unloading regulated by stress. The loading rate was 0.12 mm/min, while the unloading rate was 0.05 MPa/s. In this path, each cycle involved an axial displacement increment of 0.06 mm higher than the target displacement of the previous cycle, followed by unloading. This process was repeated until specimen failure. All compression loading paths in this study were uniaxial rather than triaxial. Figure 3 illustrates the three loading paths, where the horizontal axis represents the strain, calculated as Δε equal to 0.06 mm/100 mm = 6 × 10^−4^ for the third loading path.

To investigate the effects of the saturation level, the FRC specimens were subjected to different preconditioning methods before loading. The dried samples were placed in an oven at 65 °C for over 24 h. The saturated specimens underwent vacuum treatment at 100 kPa for more than 4 h, followed by immersion in water for 24 h. Additional specimens were tested in their natural state, referring to ambient laboratory conditions with a relative humidity of approximately 55% and a temperature of 20 °C.

## 3. Mechanical Response Under Different Loading Paths

### 3.1. Stress–Strain Behavior

**Figure 4:**
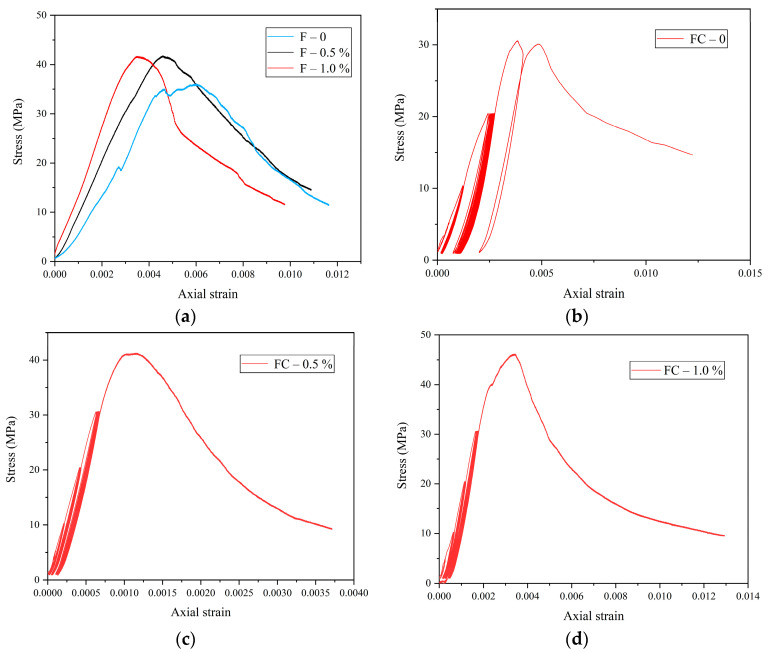

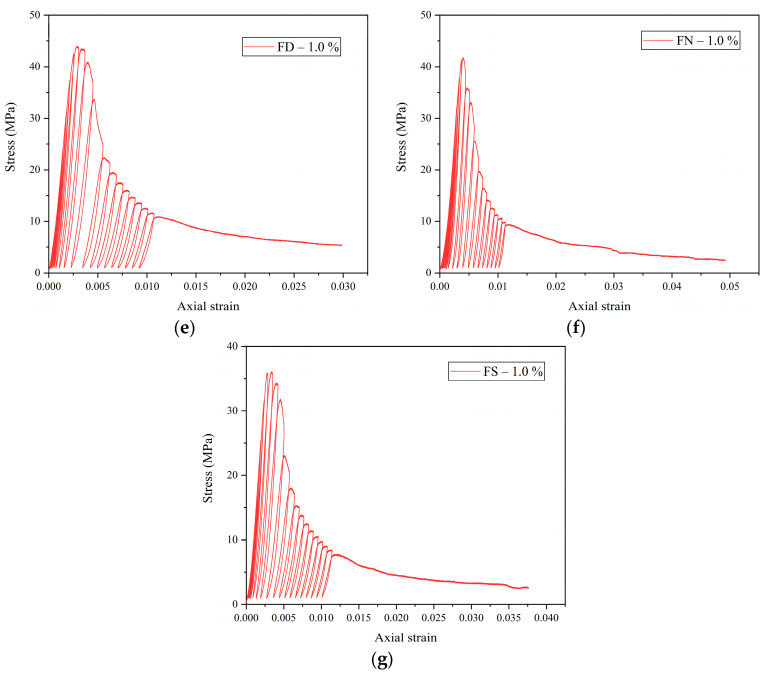
Stress–strain curves of FRC: (**a**) monotonic loading; (**b**) FC-0; (**c**) FC-0.5%; (**d**) FC-1.0%; (**e**) FD-1.0%; (**f**) FN-1.0%; (**g**) FS-1.0%.

Figure 4 shows the stress–strain curves of FRC. F represents the FRC under monotonic loading, FC represents the FRC under pre-peak cyclic loading, and FD, FN, and FS represent the dry, natural, and saturated FRC under the full cyclic loading, respectively. The 0, 0.5%, and 1.0% after the short lines represent the fiber contents. The stress–strain behavior of FRC can be categorized into five stages: (1) Compaction stage: Initial defects, microcracks, and fiber-induced air bubbles begin to close, causing a slow increase in stress. This stage is relatively short, occupying only a small portion of the pre-peak region. The critical transition between compaction and linear elastic deformation is marked by the crack closure stress. (2) Linear elastic deformation stage: The stress–strain curve exhibits linear behavior with no new crack initiation or propagation. (3) Stable crack development stage: Microcracks begin to emerge and gradually expand. (4) Crack instability propagation stage: The crack growth and intersection accelerates, leading to faster damage evolution, greater stress–strain nonlinearity, and significant plastic deformation. (5) Post-peak softening stage: A rupture surface forms, causing the specimen to fragment. This stage is characterized by a sharp stress drop, along with significant increases in the axial and circumferential strain.

During cyclic loading, the stiffness of each cycle decreases compared to the previous one. This significant stiffness degradation indicates progressive damage accumulation with each loading cycle. As shown in Figure 4e–g, the peak stress of FRC decreases with increasing saturation levels. The strength of FS-1.0% (saturated) is reduced by 17.05% compared to FD-1.0% (dry), demonstrating a pronounced water-weakening effect. This phenomenon has been widely reported in previous studies. Wu et al. [32] found that the four-point bending strength of concrete decreases with increasing saturation, while Vu et al. [33] observed a substantial reduction in the triaxial compressive strength of concrete under a high confining pressure due to the saturation. The water-weakening effect in FRC can be attributed to several factors: (1) Microcrack expansion: Pre-existing microcracks and pores provide pathways for water ingress. (2) Hydration-induced stress: Water absorption by hydration products and aggregates generates expansion stress, which enlarges the existing microcracks and triggers new ones, increasing the crack density and reducing strength. (3) Lubrication effect: The water acts as a lubricant at crack interfaces, lowering the friction between fracture surfaces and reducing the shear strength. (4) Effective stress reduction: The total stress consists of the effective stress and pore water pressure. The higher saturation level leads to increased pore water pressure, thereby reducing the effective stress and overall strength.

**Figure 5:**
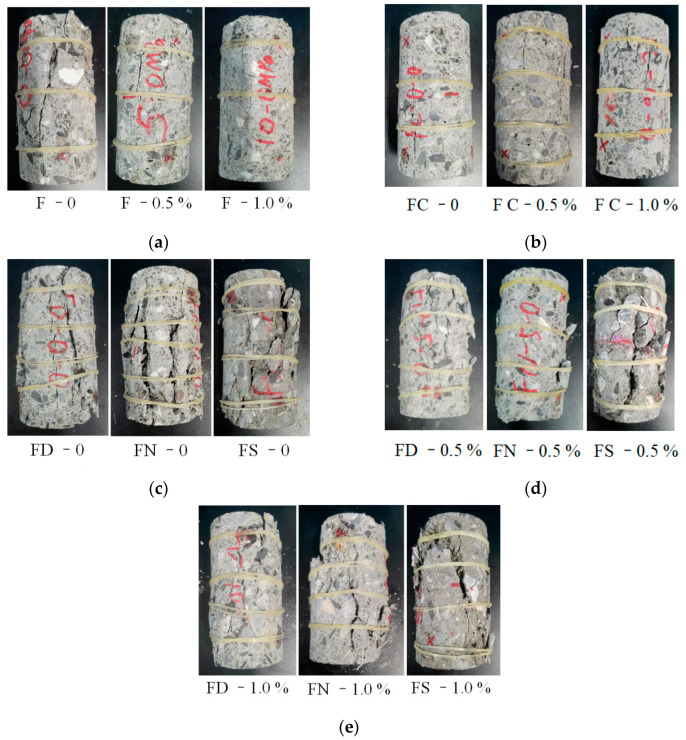
Failure patterns of FRC: (**a**) monotonic loading; (**b**) pre-peak cyclic loading; (**c**) full cyclic loading—fiber content 0; (**d**) full cyclic loading—fiber content 0.5%; (**e**) full cyclic loading—fiber content 1.0%.

Figure 5 displays the failure modes of FRC. The failure mode of FRC-1.0% is relatively intact, with fewer macroscopic cracks and fragments. The number and width of the cracks in F-1.0% are smaller than those in F-0, indicating that fiber can limit the opening of cracks. Fiber plays a crucial role in the compressive failure of concrete by hindering the crack propagation, thereby effectively enhancing the crack resistance and deformation capacity. Even after failure, FRC can still retain structural integrity and does not fragment into multiple pieces, indicating the fiber’s effectiveness in maintaining the material cohesion and bridging microcracks. Additionally, the fiber bridging effect provides extra load-bearing capacity after the concrete experiences tensile cracking, improving its ductility and fracture energy and enhancing the material’s toughness under loading. Furthermore, by reducing the crack formation, fibers mitigate the ingress of water into the concrete, thereby increasing its impermeability and long-term durability. According to Golewski’s research [34], concrete inherently contains a considerable number of initial defects, which are the primary source for microcrack propagation. As loading continues, these microcracks further propagate and coalesce, forming macroscopic dilatational cracks. In plain concrete (without fibers), the dominant failure mechanism involves tensile-induced cracking, where cracks propagate along the interface between the aggregates and the cementitious matrix. This results in the formation of striped or blocked fragmentation. Additionally, under cyclic loading, the damage in FRC becomes more severe, as evidenced by increased cracking, fragmentation, and significant circumferential strain growth. Cyclic loading causes continuous crack opening and closing, promoting damage accumulation and the progressive loosening of the internal structure. According to the failure modes of FRC with various saturations, fiber exhibits better crack bridging ability in the dry specimen, and the crack width of FD-1.0% is smaller than that of FD-0.

### 3.2. Volumetric Strain Characteristics

The equation for the volumetric strain is derived from elasticity theory, neglecting higher-order small terms [35]:

(1) $$\begin{array}{l} \varepsilon_{v}=\frac{\Delta V}{V_{0}}=\frac{V_{1}-V_{0}}{V_{0}}=\frac{V_{0}\left(1+\varepsilon_{x}\right)\left(1+\varepsilon_{y}\right)\left(1+\varepsilon_{z}\right)-V_{0}}{V_{0}}\\ \begin{array}{cc} & \begin{array}{cc} & \begin{array}{cc} & = \end{array} \end{array} \end{array}\varepsilon_{x}+\varepsilon_{y}+\varepsilon_{z}+\varepsilon_{x}\varepsilon_{y}+\varepsilon_{y}\varepsilon_{z}+\varepsilon_{z}\varepsilon_{x}+\varepsilon_{x}\varepsilon_{y}\varepsilon_{z}\\ \begin{array}{cc} & \begin{array}{cc} & \begin{array}{cc} & \approx \end{array} \end{array} \end{array}\varepsilon_{x}+\varepsilon_{y}+\varepsilon_{z}\\ \begin{array}{cc} & \begin{array}{cc} & \begin{array}{cc} & = \end{array} \end{array} \end{array}\frac{\partial u}{\partial x}+\frac{\partial v}{\partial y}+\frac{\partial w}{\partial z}\\ \begin{array}{cc} & \begin{array}{cc} & \begin{array}{cc} & = \end{array} \end{array} \end{array}\varepsilon 1+\varepsilon 2+\varepsilon 3\\ \begin{array}{cc} & \begin{array}{cc} & \begin{array}{cc} & = \end{array} \end{array} \end{array}\varepsilon 1+2\varepsilon 3 \end{array}$$

where $\varepsilon_{v}$ is the volumetric strain, ε1 is the axial strain, ε3 is the circumferential strain and is negative, ΔV is the change in volume, *V*_1_ and *V*_0_ are the volume after and before deformation, $\varepsilon_{x}$, $\varepsilon_{y}$, and $\varepsilon_{z}$ are the strain components in the *x*, *y*, and *z* directions, and *u*, *v*, and *w* are the displacement components in the *x*, *y*, and *z* directions.

**Figure 6:**
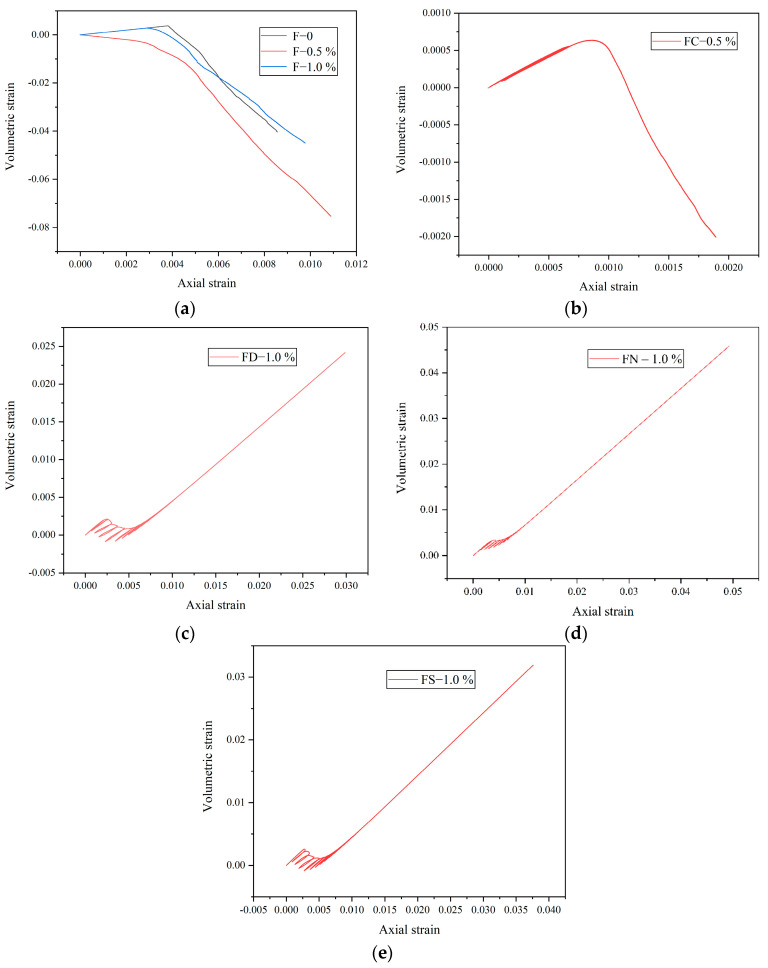
Volumetric strain–axial strain curves of FRC: (**a**) monotonic loading; (**b**) FC-0.5%; (**c**) FD-1.0%; (**d**) FN-1.0%; (**e**) FS-1.0%.

Figure 6 presents the volumetric strain–axial strain curves of FRC, where the compression is positive and the tension is negative. Figure 6a illustrates the curve under monotonic loading, showing an initial increase in the axial strain, followed by a decrease. The stress at the turning point is defined as the expansion stress, marking the transition from compression to expansion. When the volumetric strain is positive, the axial strain dominates, indicating compression. As the lateral strain increases, the volumetric strain decreases below zero, signifying that the lateral deformation governs, causing specimen expansion. Figure 6b shows that the positive volumetric strain of FC-0.5% under pre-peak cyclic loading is greater than that of F-0.5%, suggesting that pre-peak cyclic loading induces greater axial deformation. FRC with varying saturation levels exhibits distinct behavior under full cyclic loading, as shown in Figure 6c–e. In this loading path, the post-peak volumetric strain remains positive and correlates positively with the axial strain, indicating rapid axial strain development. The axial strain increment surpasses twice the circumferential strain increment, maintaining a predominantly compressive state.

### 3.3. Elastic Modulus

To compare the mechanical properties of FRC under different loading paths, the elastic modulus was calculated, providing essential data for establishing the FRC damage constitutive model. The elastic modulus was determined using equation [36]:

(2) $$E=\frac{\sigma \left(0.6\sigma_{a}\right)-\sigma \left(0.4\sigma_{a}\right)}{\varepsilon 1\left(0.6\sigma_{a}\right)-\varepsilon 1\left(0.4\sigma_{a}\right)}$$

where $\sigma_{a}$ is the peak stress and ε1 is the axial strain. The elastic modulus of the specimen was calculated using the strains corresponding to 60% and 40% of the peak stress.

**Figure 7:**
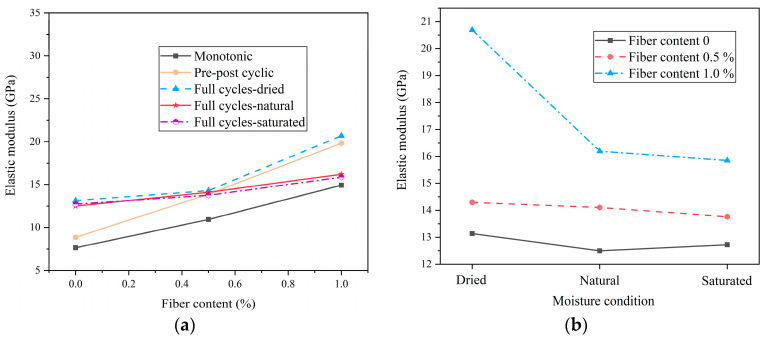
Elastic modulus of FRC: (**a**) variation in elastic modulus with fiber content; (**b**) variation in elastic modulus with moisture content.

Figure 7a illustrates the correlation between elastic modulus and fiber content across various loading paths. Compared with FRC-1.0%, the elastic modulus of FRC-0 under monotonic loading, pre-peak cyclic loading, full cyclic loading (dry state), full cyclic loading (natural state), and full cyclic loading (saturated state) decreased by 48.85%, 72.19%, 36.51%, 22.84%, and 19.73%, respectively. The results show that the deformation resistance of the FRC is improved. Figure 7b presents the relationship between the elastic modulus and the saturation level in FRC with different fiber contents. The saturation level affects the elastic modulus, with the saturated specimens exhibiting lower stiffness due to the reduced friction between cracks and the decreased resistance to deformation.

## 4. Damage Distribution Mechanism Based on AE

### 4.1. Cumulative Ring Counts

AE technology is a non-destructive monitoring method that enables the real-time tracking of material behavior by detecting and analyzing elastic waves generated by crack development. Common AE parameters include ring count, hits, amplitude, energy, intensity, and peak frequency. Among these, the ring count is widely used in AE analysis, as it dynamically reflects the intensity of AE activity and crack progression. The development of the cumulative ring count can characterize the growth of cracks and the damage evolution.

**Figure 8:**
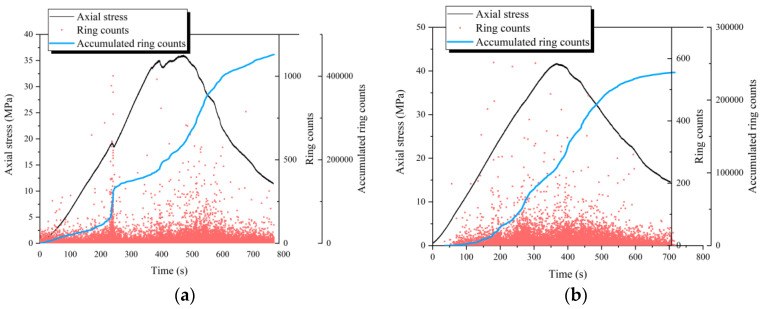

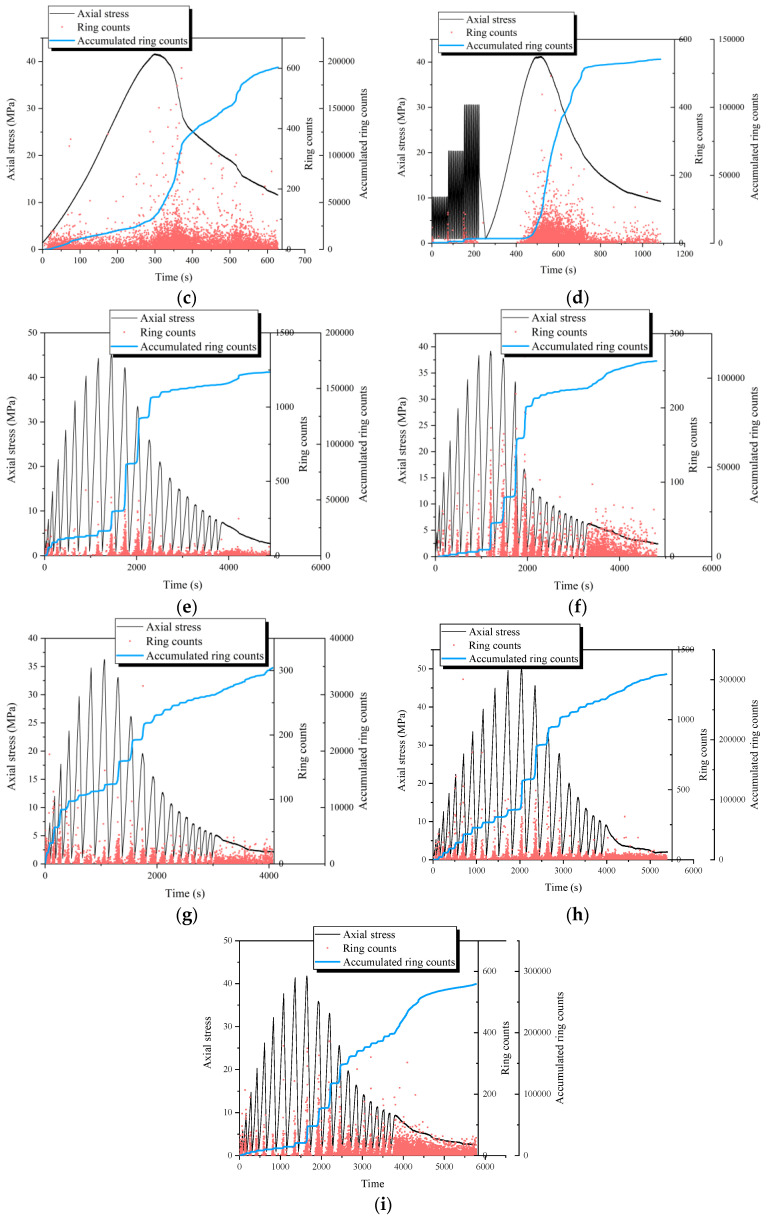
Cumulative ring count curves of FRC: (**a**) F-0; (**b**) F-0.5%; (**c**) F-1.0%; (**d**) FC-0.5%; (**e**) FD-0.5%; (**f**) FN-0.5%; (**g**) FS-0.5%; (**h**) FN-0; (**i**) FN-1.0%.

Figure 8 presents the cumulative ring count curves of FRC under various loading paths. Under monotonic loading, the ring counts are evenly distributed throughout the loading process, with a notable increase near peak stress, indicating significant crack expansion and strain energy release. The cumulative ring count curve follows a three-stage development: (1) Slow growth stage: Includes the initial compaction stage, elastic deformation stage, and stable crack propagation stage, with minimal damage. (2) Rapid growth stage: Corresponds to the crack instability propagation stage and the post-peak softening phase, where the damage increases sharply. (3) Stable stage: Occurs in the residual stage, where the damage growth stabilizes, indicating an imminent specimen failure. Under pre-peak cyclic loading (Figure 8d), the ring counts concentrate near peak stress, with each increase in the upper-stress limit triggering a surge in AE signals. This suggests that the first cycle at each stress level initiates new microcracks and damage, while the following 14 cycles primarily involve repetitive crack opening and closing with minimal additional damage. The cumulative ring count curve confirms that pre-peak cyclic loading induces limited damage before peak stress, after which the crack propagation accelerates rapidly, leading to rupture surface formation, with a sharp surge in AE activity. Conversely, under full cyclic loading (Figure 8e–g), each cycle induces a sharp increase in the ring counts, indicating that every cycle contributes to new plastic deformation. Consequently, the cumulative ring count curve exhibits a stepwise increase. Figure 8f,h,i indicate that the pre-peak growth rate of the cumulative ring count curves for FN-1.0% and FN-0.5% is significantly lower than that of FN-0. This suggests that fibers effectively retard crack extension and damage accumulation during pre-peak loading and unloading.

Moreover, the pre-peak cumulative ring count curve of the dry and natural FRC evolves more gradually than that of the saturated FRC. The damage in the saturated FRC escalates more rapidly, further substantiating the water-weakening effect in FRC. These findings highlight the significant role of the saturation level in damage progression and underscore the importance of accounting for the water-weakening effect in the design of durable FRC structures exposed to wet environmental conditions.

### 4.2. Spatial Distribution of Fracture Damage Based on Logistic Regression

**Figure 9:**
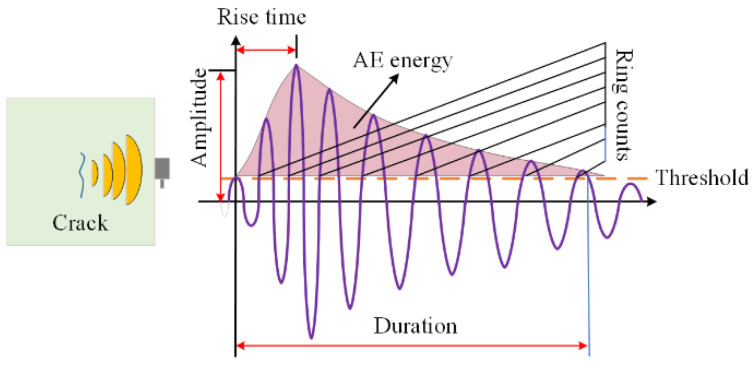
AE signal waveform.

Cracks in compression-damaged concrete can be classified into tensile and shear damage, which can be identified using the AF-RA method. A high RA value and a low AF value indicate shear cracks, whereas a high AF value and a low RA value suggest tensile cracks [37]. To analyze these crack modes, Figure 9 presents the AE signal waveform, where AF is defined as the ratio of ring counts to duration, and RA is the ratio of rise time to signal amplitude [38].

Many studies have proposed specific functions as benchmarks for AF-RA classification. However, this classification is not universally applicable across all materials, and various discriminant functions have been developed in the literature to address different materials, making it challenging to establish a unified criterion. To classify the AF-RA distributions, a binary classification approach based on the logistic regression (LR) algorithm is employed to distinguish the different compression damage types of FRC under various loading conditions. The LR algorithm is a generalized linear model commonly used for the binary or multi-class classification of fractional datasets. After training, the model essentially represents a straight line in a two-dimensional plane, a plane in three-dimensional space, or a hyperplane in higher dimensions. The mean square error function is adopted to evaluate the accuracy of the algorithm training results [39]:

(3) $$J\left(w\right)=\frac{1}{n}\sum_{i=1}^{n}\frac{1}{2}{\left(h\left(x_{i}\right)-y_{i}\right)}^{2}$$

where *h*(*x_i_*) is the model predicted value and *y_i_* is the sample actual value.

**Figure 10:**
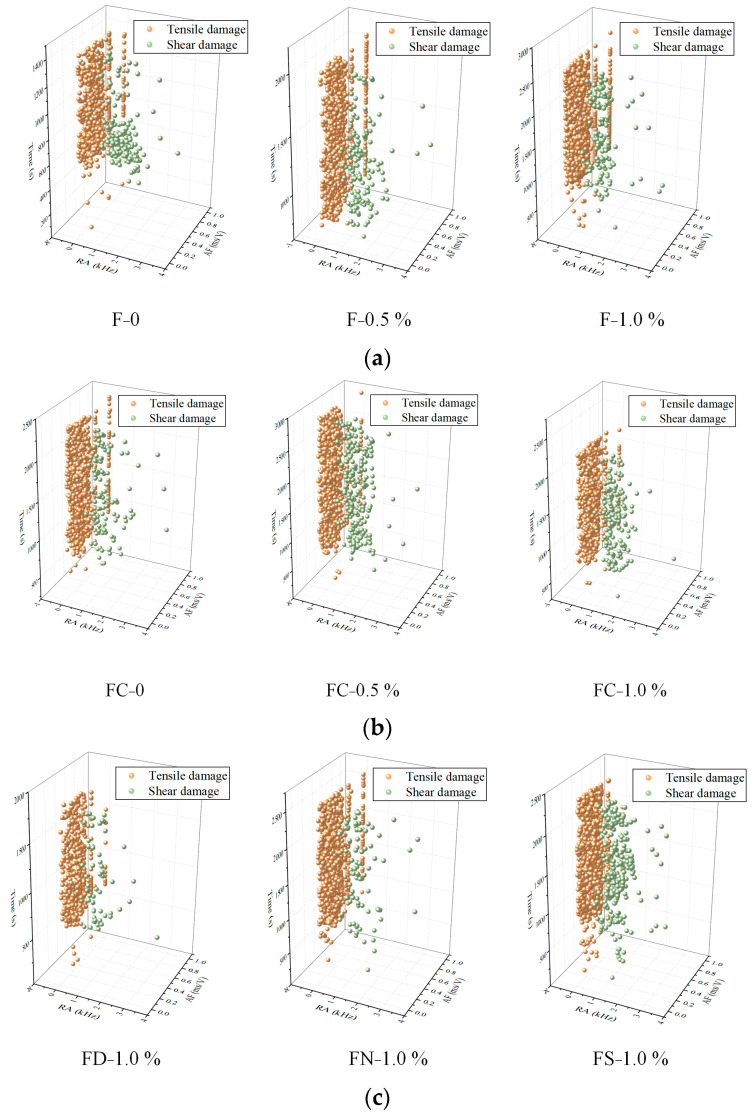
Damage types of FRC under different conditions: (**a**) monotonic loading; (**b**) pre-peak cyclic loading; (**c**) full cyclic loading.

Before applying the LR algorithm for training and prediction, AF and RA data must be standardized. Figure 10 illustrates the classification of the FRC damage types under different loading conditions based on the AF-RA analysis, with the model achieving an accuracy of 95.7%. The frequency of shear damage signals in FRC subjected to various fiber contents and loading paths is significantly lower than that of tensile damage signals. The failure mode is predominantly tensile, with tensile damage signals appearing from the beginning of loading to the final rupture.

**Figure 11:**
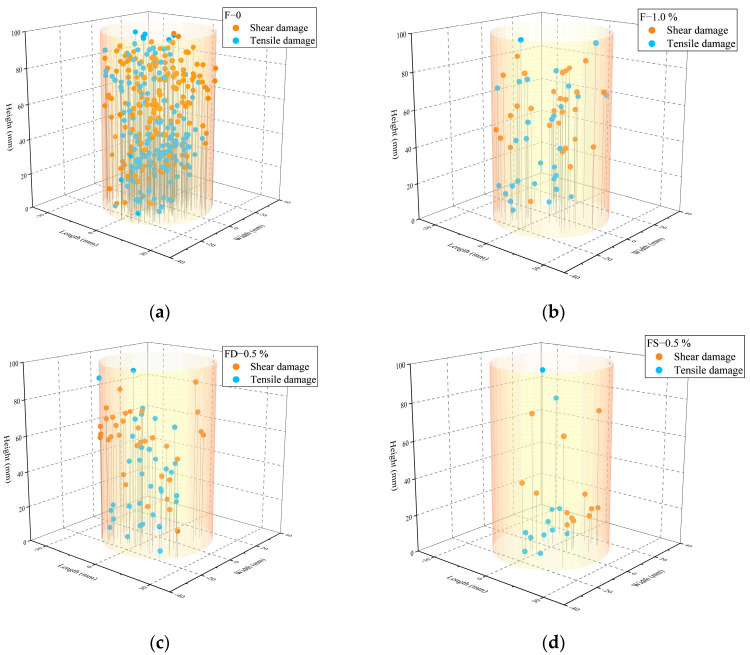
AE event classification: (**a**) F-0; (**b**) F-1.0%; (**c**) FD-1.0%; (**d**) FS-1.0%.

An AE event refers to a distinct AE activity detected through multiple hits. The spatial coordinates of AE events provide insights into the internal damage distribution within FRC, and are determined based on the acoustic wave propagation time. Notably, at least four sensors are required for accurate AE event localization. Based on the AF-RA classification, the corresponding RA distributions for the tensile and shear damages are obtained. These distributions are then used to categorize the AE event coordinates, visualizing the spatial distribution of the damage types, as shown in Figure 11. The orange dots represent shear damage, while the blue dots indicate tensile damage. Across the various fiber and saturation levels, the tensile damage events significantly outnumber the shear damage events, further confirming that the dominant failure mode of FRC under different loading paths is tensile failure.

**Figure 12:**
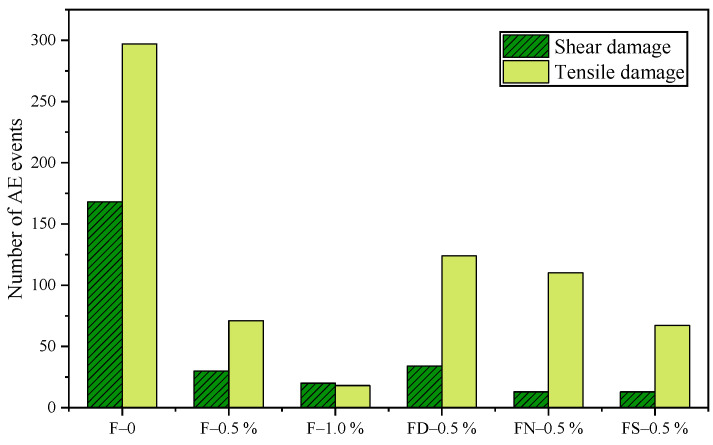
AE events: shear–tensile damage distribution.

Expanding on the AF-RA classification obtained through the LR analysis, further identification of the AE damage types was conducted. To better illustrate the effects of the saturation level and fiber content on the FRC damage types, Figure 12 presents the shear–tensile damage distribution of AE events. The total number of AE events in the FRC specimens with 0.5% and 1.0% fiber content is notably lower than in F-0, indicating more intense AE activity in F-0. As the fiber content increases, the proportion of tensile damage events decreases significantly, demonstrating the crack bridging effect of fibers. Li et al. [21] reported similar findings, indicating that failure in SFRC is primarily associated with shear cracking due to fiber pull-out and sliding. Their study also showed that the frequency of fiber pull-out and sliding events increases with the fiber volume fraction and aspect ratio, which aligns with the experimental results of this study.

Additionally, when comparing the dry (FD-0.5%), natural (FN-0.5%), and saturated (FS-0.5%) specimens, the total number of AE events decreases with increasing saturation levels. However, the saturation level does not significantly affect the relative proportions of shear and tensile damage.

### 4.3. Gaussian Process Regression of AE Multi-Parameters

A Gaussian process (GP) is a stochastic process characterized by the joint probability density distribution of a set of random variables, extending the concept of multivariate Gaussian distributions. These random variables are continuously distributed over time or space and follow a multivariate normal distribution. A one-dimensional Gaussian distribution is typically expressed as [40,41]:

(4) $$p\left(x\right)=\frac{1}{\sigma \sqrt{2\pi }}\exp\left(\frac{-{\left(x-\mu \right)}^{2}}{2\sigma^{2}}\right)$$

where *μ* is the average value and σ is the variance.

For a multi-dimensional Gaussian distribution, assuming independence between the dimensions, the probability density function is given by the following:

(5) $$\begin{array}{l} p\left(x_{1},x_{2},\;\ldots \;,\;x_{n}\right)=\prod_{i=1}^{n}p\left(x_{i}\right)\\ \;=\frac{1}{{\left(2\pi \right)}^{\frac{n}{2}}\sigma_{1}\sigma_{2\ldots }\sigma_{n}}\exp\left(-\frac{1}{2}\left[\frac{{\left(x_{1}-\mu_{1}\right)}^{2}}{\sigma_{1}^{2}}+\frac{{\left(x_{2}-\mu_{2}\right)}^{2}}{\sigma_{2}^{2}}+\ldots +\frac{{\left(x_{n}-\mu_{n}\right)}^{2}}{\sigma_{n}^{2}}\right]\right) \end{array}$$

The equation can be expressed as the vector and matrix:

(6) $$\begin{array}{l} \vec{x}-\vec{\mu }={\left[x_{1}-\mu_{1},x_{2}-\mu_{2},\;\ldots \;,xn-\mu_{n}\right]}^{T}\\ G=\left[\begin{array}{cccc} \sigma_{1}^{2} & 0 & \cdots & 0\\ 0 & \sigma_{2}^{2} & \cdots & 0\\ \vdots & \vdots & \ddots & \vdots \\ 0 & 0 & \cdots & \sigma_{n}^{2} \end{array}\right] \end{array}$$

(7) $$\begin{array}{l} \sigma_{1}\sigma_{2\ldots }\sigma_{n}={\left|G\right|}^{\frac{1}{2}}\\ \frac{{\left(x_{1}-\mu_{1}\right)}^{2}}{\sigma_{1}^{2}}+\frac{{\left(x_{2}-\mu_{2}\right)}^{2}}{\sigma_{2}^{2}}+\ldots +\frac{{\left(x_{n}-\mu_{n}\right)}^{2}}{\sigma_{n}^{2}}={\left(\vec{x}-\vec{\mu }\right)}^{T}G^{-1}\left(\vec{x}-\vec{\mu }\right) \end{array}$$

Substituting the Equation (7) into (5) gives

(8) $$p\left(x\right)={\left(2\pi \right)}^{-\frac{n}{2}}{\left|G\right|}^{-\frac{1}{2}}\exp\left(-\frac{1}{2}{\left(\vec{x}-\vec{\mu }\right)}^{T}G^{-1}\left(\vec{x}-\vec{\mu }\right)\right)$$

where $\vec{\mu }$ is the mean value vector, *G* is the covariance matrix, and $x∼N\left(\vec{\mu },G\right)$.

Since a Gaussian process is a random process on a continuous field, a Gaussian process can be expressed as follows:

(9) $$f\left(\vec{x}\right)∼N\left(\mu \left(x\right),G\left(x,x\right)\right)$$

where $\mu \left(x\right)$ is the mean value function and *G* (*x*, *x*) is the covariance function, that is, the kernel function.

Gaussian process regression (GPR) builds upon the GP framework by incorporating observed data to refine the mean and kernel functions, enabling a regression analysis of the sample datasets. A commonly used Gaussian kernel function takes the form

(10) $$G\left(x_{i},x_{j}\right)=\sigma^{2}\exp\left(-\frac{{\left\|x_{i}-x_{j}\right\|}_{2}^{2}}{2l^{2}}\right)$$

where σ and *l* are hyper-parameters, which are crucial for the optimization of the GPR results.

**Figure 13:**
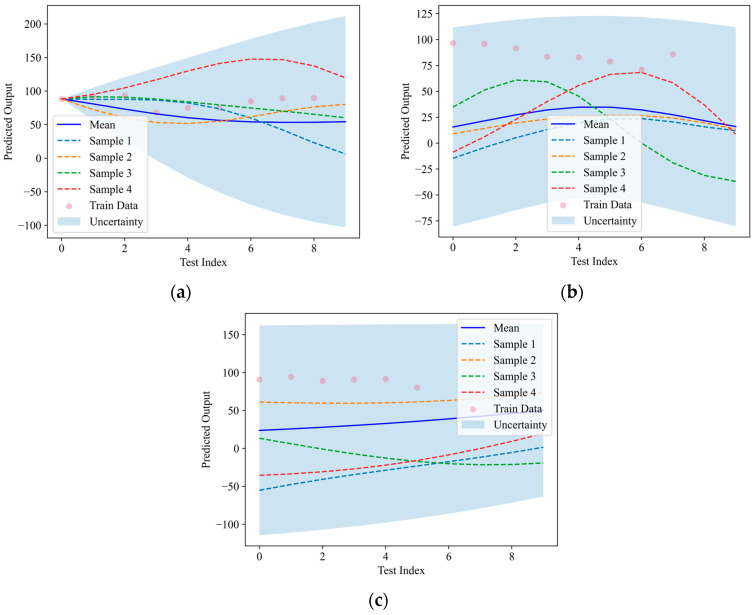
One-dimensional predictions of AE tensile damage parameters based on GPR: (**a**) monotonic loading; (**b**) pre-peak cyclic loading; (**c**) full cyclic loading.

**Figure 14:**
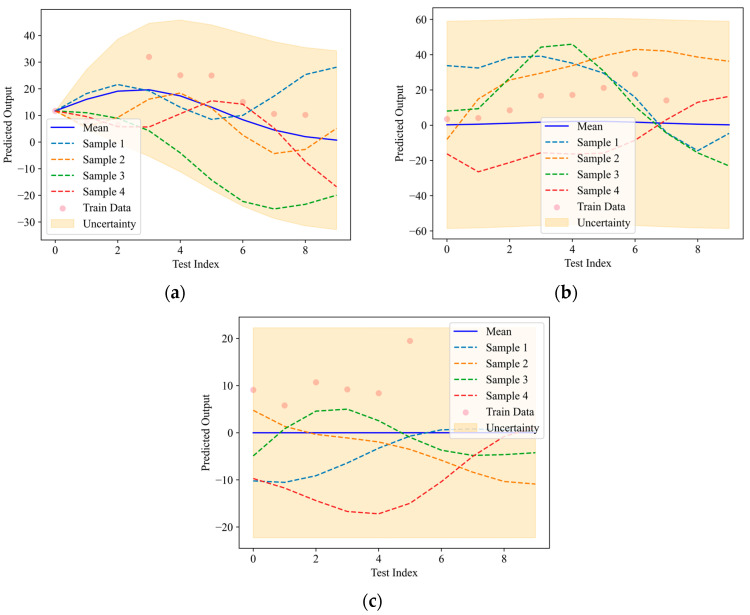
One-dimensional predictions of AE shear damage parameters based on GPR: (**a**) monotonic loading; (**b**) pre-peak cyclic loading; (**c**) full cyclic loading.

To analyze the AE characteristics in FRC under compression, GPR was applied to estimate the 95% confidence intervals for the damage proportion regression under different loading conditions. Initially, the one-dimensional input variables—fiber content and saturation level—were used as the input features, with the tensile damage and shear damage serving as the response parameters. Figure 13 and Figure 14 illustrate the one-dimensional GPR predictions for the AE tensile and shear damage parameters in FRC, respectively. In addition to the predicted values and confidence intervals, four randomly selected sampling curves are included to represent the potential variations within the confidence range.

**Figure 15:**
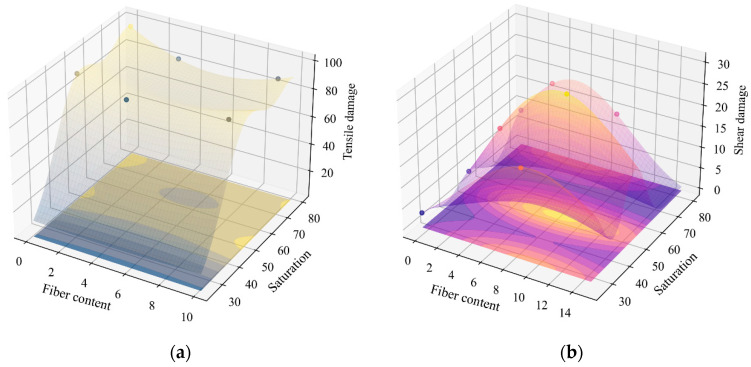
Multi-dimensional predictions of AE damage parameters based on GPR: (**a**) tensile damage; (**b**) shear damage.

Expanding the input dimensions to include both fiber content and saturation level as the input variables, multi-dimensional GPR predictions were generated. Figure 15 presents the multi-dimensional GPR results for the AE tensile and shear damage parameters in FRC subjected to full cyclic loading. The wave crest in the response surface indicates the predicted extreme values of the damage parameter under specific fiber content and saturation levels. The locations and magnitudes of these peaks reflect the extent of the damage under varying conditions, providing insight into the different responses of FRC under diverse environmental and mechanical conditions.

## 5. Damage Constitutive Model

Based on continuum damage mechanics and the Lemaitre equivalent strain principle, the strain of a damaged material under nominal stress (i.e., Cauchy stress) can be considered equivalent to the strain of an undamaged material under effective stress. Sidoroff [42] introduced the energy equivalence principle, which states that by replacing the nominal stress tensor with the effective stress tensor, the elastic residual energy corresponding to the true stress–strain behavior of a damaged material can be characterized as the elastic residual energy of an undamaged material subjected to effective stress. This concept is known as the elastic residual energy equivalence principle. The damage factor (*D*) is expressed as follows:

(11) $$D=1-\sqrt{E/E_{0}}$$

This is equivalent to the below equation

(12) $$E={\left(1-D\right)}^{2}E_{0}$$

Then, the stress softening relationship is as follows:

(13) $$\begin{array}{l} \sigma_{e}=E_{0}\varepsilon \\ \sigma ={\left(1-D\right)}^{2}\sigma_{e} \end{array}$$

where *E*_0_ is the elastic modulus of the elastic stage, $\sigma_{e}$ is the nominal elastic stress, and *E* and σ are the elastic modulus and stress of the softening section.

According to the damage evolution equation derived from the energy equivalence principle proposed by Ghrib and Tinawi [43],

(14) $$\left\{\begin{array}{l} D=0,\\ D=1-\sqrt{\frac{\varepsilon_{0}}{\varepsilon^{*}}\left(2e^{-b\left(\varepsilon^{*}-\varepsilon 0\right)}-e^{-2b\left(\varepsilon^{*}-\varepsilon 0\right)}\right)}, \end{array}\right.\begin{array}{c} \varepsilon^{*}\leq \varepsilon_{0}\\ \begin{array}{l} \varepsilon^{*}>\varepsilon_{0} \end{array} \end{array}$$

(15) $$b=\frac{3}{\varepsilon_{0}\left(\frac{2G_{f}E}{l_{\mathrm{ch}}f_{t}^{2}}-1\right)}\geq 0$$

where *G_f_* is the fracture energy of the concrete, and *l*_ch_ is the characteristic length, which is related to the aggregate size.

Combined with Equations (11) and (14), in the softening section,

(16) $$\sqrt{\frac{E}{E_{0}}}=\sqrt{\frac{\varepsilon_{0}}{\varepsilon^{*}}\left(2e^{-b\left(\varepsilon^{*}-\varepsilon 0\right)}-e^{-2b\left(\varepsilon^{*}-\varepsilon 0\right)}\right)}$$

Because $f_{c}=E_{0}\varepsilon_{0}$, this converts to the following:

(17) $$E=\frac{f_{c}}{\varepsilon^{*}}\left(2e^{-b\left(\varepsilon^{*}-\varepsilon 0\right)}-e^{-2b\left(\varepsilon^{*}-\varepsilon 0\right)}\right)$$

Then, the equivalent stress is as follows:

(18) $$\sigma *=f_{c}\left(2e^{-b\left(\varepsilon^{*}-\varepsilon 0\right)}-e^{-2b\left(\varepsilon^{*}-\varepsilon 0\right)}\right)$$

Therefore, the damage evolution follows an exponential stress–strain relationship in the softening phase:

(19) $$\left\{\begin{array}{l} \sigma =E_{0}\varepsilon ,\\ \sigma =f_{c}\left\{2\exp\left[-b\left(\varepsilon -\varepsilon_{0}\right)\right]-\exp\left[-2b\left(\varepsilon -\varepsilon_{0}\right)\right]\right\}, \end{array}\right.\begin{array}{c} \varepsilon \leq \varepsilon_{0}\\ \begin{array}{l} \varepsilon >\varepsilon_{0} \end{array} \end{array}$$

**Figure 16:**
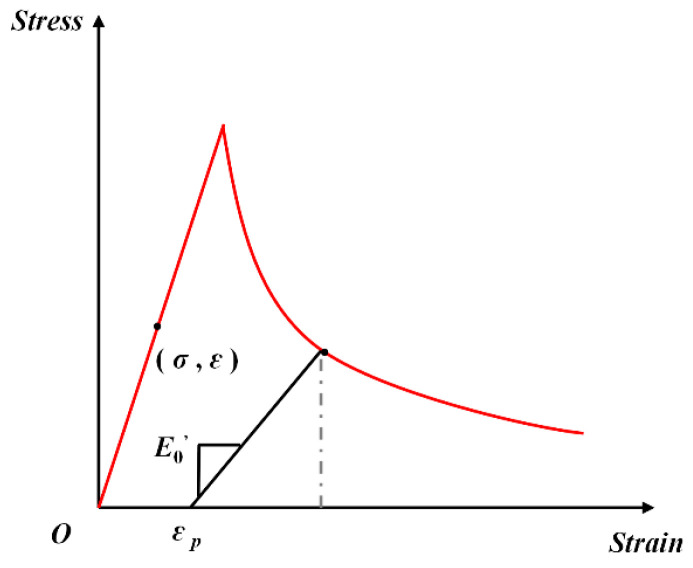
The diagram of the stress–strain skeleton.

The diagram of the stress–strain skeleton is shown in Figure 16.

**Figure 17:**
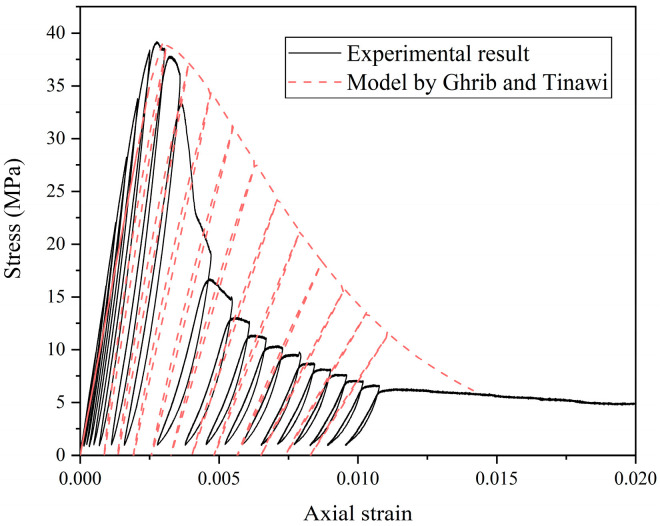
Experimental results and theoretical model of stress–strain curve (FN-0.5%).

Using the experimental data, the stress–strain curve of FN-0.5% was fitted with this model, and the results are presented in Figure 17. In the pre-peak phase, the material exhibits near-elastic behavior, closely aligning with the monotonic loading envelope. The plastic strain in this phase is minimal and can be neglected. However, in the post-peak region, the degradation of the elastic modulus should be considered. The post-peak plastic strain is calculated as the difference between the strain at each unloading point and the strain at the intersection of the unloading curve and the strain axis. As the stress begins to decrease, the strain values are recorded, and Equation (16) is used to compute the degradation of the elastic modulus for each cycle. This degradation modulus is then employed to determine the strain when the stress decreases to zero, thereby obtaining the plastic strain for each cycle. The damage variable *D* for each unloading step is computed using Equation (14) and then substituted into Equation (12) to determine the attenuated elastic modulus.

While the model proposed by Ghrib and Tinawi accounts for the stiffness degradation during unloading, it does not accurately capture the post-peak softening behavior of FRC. This is evident from the discrepancies between the model’s predictions and the experimental results. Therefore, a new post-peak softening model is needed to better describe the mechanical response of FRC. The proposed model is formulated as follows:

(20) $$\left\{\begin{array}{l} \sigma =E_{0}\varepsilon ,\\ \sigma =f_{c}\left\{1-k\left[\varepsilon_{un}\left(\varepsilon -\varepsilon_{0}\right)/\left(\varepsilon \left(\varepsilon_{un}-\varepsilon_{0}\right)\right)\right]\right\}, \end{array}\right.\begin{array}{c} \varepsilon \leq \varepsilon_{0}\\ \begin{array}{l} \varepsilon >\varepsilon_{0} \end{array} \end{array}$$

where $\varepsilon_{un}$ represents the failure strain and *k* is a material-dependent parameter. The parameter *k* influences the shape and steepness of the post-peak softening curve and is affected by material properties such as strength, saturation level, and brittleness. For high-strength and brittle materials, *k* tends to be larger, whereas for materials with higher water content, *k* is smaller. In triaxial compression tests, *k* also depends on the testing conditions, such as the confining pressure, and tends to decrease under higher confinement. In this study, the selected range for *k* is between 0.6 and 1.1, making it a dimensionless semi-empirical parameter.

**Figure 18:**
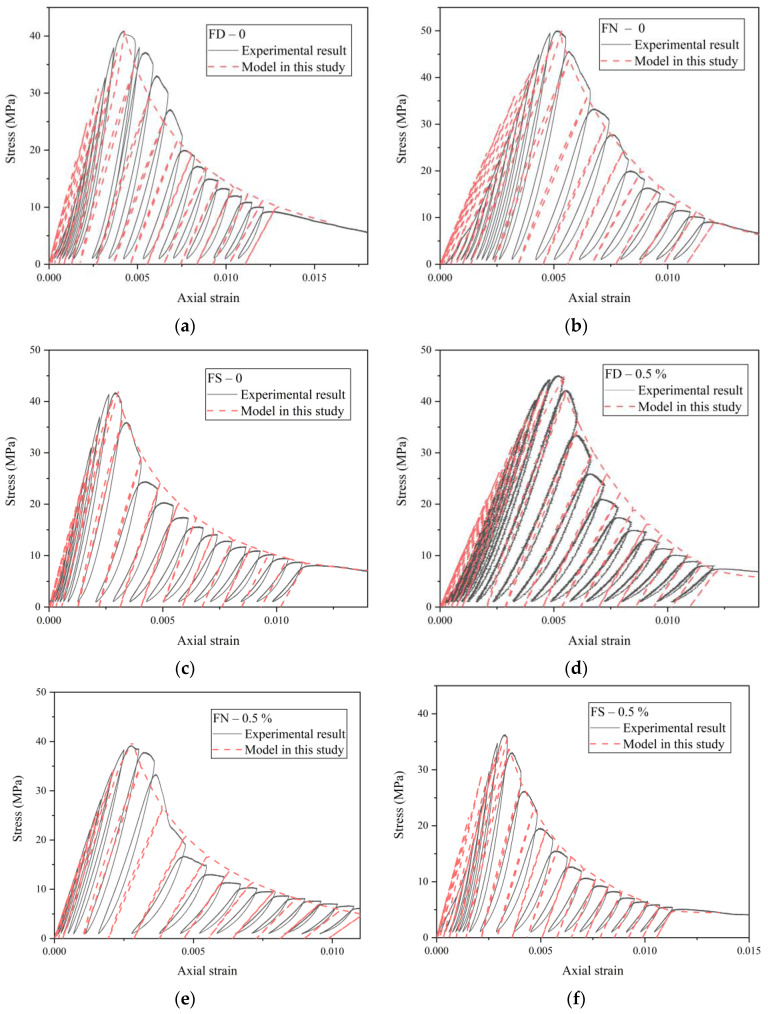
Validation of damage constitutive model: (**a**) FD-0; (**b**) FN-0; (**c**) FS-0; (**d**) FD-0.5%; (**e**) FN-0.5%; (**f**) FS-0.5%.

The proposed model was validated against the stress–strain curves of FRC under different loading conditions, as shown in Figure 18. The model demonstrated strong agreement with the experimental data and effectively captured the post-peak softening behavior of FRC, as well as the influence of the saturation level on the mechanical performance. The procedure for implementing full cyclic loading in the model is outlined as follows:

(1) Initialization: Define an initial strain ε, an initial stress σ, and a target axial strain εT.

(2) Loading Step: Given a strain increment Δε, calculate the corresponding stress σ+Δσ at the strain ε+Δε. Update the initial strain ε to ε+Δε and the initial stress σ to σ+Δσ for the next calculation step. Repeat this process *n* times until the strain reaches the target value $\varepsilon_{T}$, then initiate unloading.

(3) Unloading Step: Unloading is controlled by the axial stress while accounting for stiffness degradation. Given a stress decrement Δσ’, calculate the strain $\varepsilon_{T}-\Delta \sigma ’/E_{\deg}$ at the reduced stress σ−Δσ’ until the stress reaches the target unloading value. Reloading follows the same procedure. Steps (2) and (3) constitute one loading–unloading cycle.

(4) Cycle Completion: Repeat Steps (2) and (3) until the target number of cycles is completed. The pre-peak cyclic loading follows a similar procedure.

## 6. Conclusions

In this study, monotonic and cyclic compression tests were conducted on FRC specimens with varying fiber and saturation levels. Simultaneous AE monitoring was performed to analyze the mechanical responses and AE characteristics of FRC. The key findings are summarized as follows:

(1) Fiber improves the stiffness of FRC, the post-peak stiffness decay phenomenon of FRC under cyclic loading is obvious, and the peak stress and elastic modulus of FRC decrease with an increase in water content, exhibiting a significant water-weakening effect.

(2) Under monotonic and pre-peak cyclic compression, the volumetric strain of FRC undergoes a transition from a compressive state to an expansive deformation, with the turning point corresponding to the dilatational stress. However, under full cyclic loading, the volumetric strain of FRC at different saturation levels remains positive, indicating that the specimen primarily experiences a compressive state. This suggests that under full cyclic loading, the internal damage accumulation and structural loosening are more pronounced, leading to a more significant axial strain development.

(3) The cumulative AE ring count curve effectively describes the AE activity intensity and damage evolution in FRC, which is strongly correlated with the loading path. Under cyclic loading, each reloading phase causes a surge in AE activity, indicating microcrack propagation and damage accumulation. The cumulative ring count curve follows a distinct three-stage development (slow growth, sharp increase, and plateau), with the initial damage evolution being slower in dry FRC than in saturated FRC.

(4) The LR algorithm, a generalized linear regression and classification model, identifies tensile and shear damage based on AF-RA distribution. The frequency of the shear damage signals in FRC across different fiber contents and loading paths is significantly lower than that of the tensile damage signals, indicating that tensile failure is the dominant mode. Tensile damage signals appear throughout the loading process until the final rupture. Additionally, increasing fiber content reduces the proportion of tensile damage events, indicating that fibers mitigate tensile crack development.

(5) The GPR predictions of the AE parameters reveal the responses of tensile and shear damage under different fiber and water content levels. The location and magnitude of the predicted wave crest represent the extreme damage value, which is more pronounced under high saturation.

(6) A damage constitutive model, developed based on continuum damage mechanics and the Lemaitre equivalent strain principle, accurately characterizes the post-peak softening behavior of FRC with different fiber and saturation levels, as validated by the experimental results.

## Figures and Tables

**Table 1:** Mix proportions of FRC.

Number	Cement (kg/m^3^)	Coarse Aggregates (kg/m^3^)	Sand (kg/m^3^)	Fiber (kg/m^3^)	Water (kg/m^3^)	Superplasticizer (kg/m^3^)
FRC-0	430	705	1015	0	215	1.14
FRC-0.5%	430	700	1007	4.55	215	1.14
FRC-1.0%	430	694	999	9.1	215	1.14

**Table 2:** Properties of polypropylene fiber.

Material	Shape	Nominal Length (mm)	Equivalent Diameter (mm)	Density (g/cm^3^)	Fracture Strength (MPa)	Initial Modulus (GPa)	Origin Place
Polypropylene	Oblate	30	0.81	0.91	474	6.0	Jiangsu

**Table 3:** Properties of superplasticizer.

Water Reducing Rate (%)	Solid Content (%)	Water Bleeding Ratio (%)	Gas Content (%)	Setting Time - Difference (min)		Compressive Strength Ratio (%)	
				Initial Set	Final Set	3d	28d
29.5	24.5	30.0	1.4	+120	+197	29.5	24.5

**Table 4:** Properties of cement.

Specific Surface Area (m^2^/kg)	Setting Time (min)		Compressive Strength (MPa)		Flexural Strength (MPa)	
	Initial Set	Final Set	3d	28d	3d	28d
359	217	322	26.7	47.4	5.9	8.9

**Table 5:** Properties of coarse aggregate.

Water Absorption (%)	Voidage (%)	Apparent Density (kg/m^3^)	Silt Content (%)	Lump Content (%)
0.86	43.0	2720	0.9	0

## Data Availability

The raw data supporting the conclusions of this article will be made available by the authors on request.
